# A randomized controlled trial of telemedicine and e-health interventions for the management of oral complications in oncology patients undergoing antineoplastic therapy

**DOI:** 10.3389/fonc.2026.1774001

**Published:** 2026-05-04

**Authors:** Pinelopi Petropoulou, Ioli Ioanna Artopoulou, Ioannis Kalemikerakis, Antonis Galanos, Ourania Govina

**Affiliations:** 1 Department of Nursing, University of West Attica, Athens, Greece; 2 Department of Dentistry, National and Kapodistrian University of Athens, Athens, Greece; 3 Biostatistician, Laboratory for Research of the Musculoskeletal System (LRMS), School of Medicine National and Kapodistrian University of Athens, KAT General Hospital, Athens, Greece

**Keywords:** antineoplastic therapy, cancer, e-health, oncology patients, oral complications, oral health management, randomized controlled trial, telemedicine

## Abstract

**Background:**

Severe oral complications during and after antineoplastic therapies, such as mucositis, fungal, viral, and bacterial infections, xerostomia, dysphagia, and jaw osteonecrosis, significantly impair oral health and quality of life in oncology patients. Dentist involvement throughout oncology care is essential to reduce complications and support patient well-being. Telemedicine and e-health tools offer effective approaches for oral-health monitoring during treatment.

**Objective:**

To evaluate the effectiveness of a comprehensive dental intervention for the assessment and management of oral health in patients with solid tumors during cancer therapy using e-health applications, compared with standard oncology-based oral care.

**Methods:**

This prospective randomized interventional study was conducted at an Oncology Hospital in Athens (March 2022–November 2023), including 105 patients with breast, lung, or colon cancer initiating chemotherapy or combined therapy. Three clinical and radiographic oral-health and quality-of-life assessments were performed. The control group received standard recommendations to visit a dentist, while the intervention group followed a structured, individualized oral health - management protocol via telemedicine and clinical monitoring.

**Results:**

Telemedicine significantly improved oral-health outcomes and oral-care behaviors compared with controls. At mid-treatment and treatment completion, the intervention group showed statistically significant lower rates of mucosal lesions, gingival bleeding, gingivitis, dental caries, periodontitis, and higher tooth-brushing frequency. These results indicate that Telemedicine and e-health enable effective monitoring, early-stage detection, and oral self-care guidance, improving oral health and well-being during and after treatment.

**Conclusion:**

Integration of structured teledentistry monitoring into oncology care was associated with improved oral health outcomes in patients undergoing antineoplastic therapy, compared with usual practice.

## Introduction

In the general population, oral health is influenced by a variety of risk factors, including poor oral hygiene, periodontal diseases, dental caries, xerostomia, inadequate diet, smoking, and polypharmacy. In oncology patients, the administration of chemotherapy, radiotherapy, or targeted therapies further increases the risk of developing oral complications such as mucositis, xerostomia, dysgeusia, fungal infections, and periodontal deterioration. Therefore, oral health—although vital for every individual—acquires particular importance for cancer patients, as it directly affects both the continuity of their treatment and their health-related quality of life (HRQoL). Understanding this relationship is essential for developing effective strategies for prevention, early intervention, and interdisciplinary care (1, 2). The prevalence of major oral diseases continues to increase globally alongside rising urbanization, driven by changes in living conditions and dietary patterns, socioeconomic disparities, and inequalities in access to healthcare services. When assessing oral health in oncology patients, clinicians carefully and systematically examine for pain and fissures at the corners of the mouth, abnormal lip texture or coloration, painful, swollen, or inflamed areas on the tongue, abnormalities in the appearance or palpation of the buccal mucosa, palate, or oropharynx, salivary gland dysfunction, halitosis, altered taste or swallowing, reduced salivary flow, and xerostomia or thickened saliva (3, 4).

Additional findings may include swollen, erythematous gingiva that bleed easily on brushing, as well as loose or fractured teeth, exposed roots requiring extraction, dental pain or sensitivity, difficulties with eating and speech, poor oral hygiene, carious lesions, defective restorations, ill-fitting prostheses, and malocclusion or temporomandibular joint dysfunction. The oral cavity is recognized as the most common site of infection in immunocompromised cancer patients (5, 6).

The profound and widespread effects of cancer therapies on the oral cavity, both during and after treatment, have now been internationally acknowledged. The World Dental Federation (FDI) and the oncology community consider oral care for cancer patients an “urgent need” and a public health concern, promoting programs involving dentists, oncologists, nurses, and other health professionals. The need is particularly critical in the context of newer biological therapies, where periodontal and gingival health is linked to the development of severe complications. The involvement of a dentist in the oncology care team at all stages of patient management is essential and contributes to improved therapeutic outcomes and better quality of life (7). The goal should be to maintain oral health stability throughout antineoplastic treatment to ensure uninterrupted and timely completion of therapy. Oral complications are common during antineoplastic therapy and share many characteristics regardless of cancer type (8). In some cases, severe oral complications may significantly impact cancer treatment, necessitating dose reductions, alterations in treatment schedules, delays, or even discontinuation. Thus, before treatment initiation, patients must understand the causes of oral complications and how to manage them to reduce symptoms and improve their quality of life (9).

The severity of adverse effects appears to depend on both individual susceptibility and the cancer therapy itself. Chemotherapy can impair the proliferation of normal oral epithelial cells and reduce their ability to repair, leading to oral ulcers. It can also decrease white blood cell counts, increasing susceptibility to infection. Chemotherapy has also been shown to disrupt the oral microbiome, and such imbalances may predispose to oral disease and hinder the body’s ability to combat bacterial, viral, and fungal infections (10).

Most chemotherapy-induced oral complications are short-term and typically resolve after treatment completion. The principal oral complications described during cancer therapy include candidiasis, mucositis, herpes simplex virus infection, bacterial infections of the mucosa and dentoalveolar tissues, dry mouth, osteonecrosis of the jaws. Clinically, these manifest as mucosal inflammation and ulceration, causing pain and increasing the risk of infection; oral bleeding may occur due to thrombocytopenia (11, 12). Gingivitis, periodontitis, carious teeth, defective restorations, inappropriate prosthetic work, teeth requiring extraction, and incomplete endodontic treatments may serve as sources of localized infection that can progress to systemic involvement; therefore, they should be managed before cancer therapy begins (13). Malnutrition or dehydration may arise when patients are unable to eat or drink due to oral ulcers, xerostomia, pain, dysgeusia, or dysphagia (14, 15).

Good oral hygiene before and during cancer treatment can help prevent or reduce treatment-related oral complications. Patients should be educated on proper dental care to minimize adverse effects and manage symptoms. The aim is to address existing oral problems prior to cancer treatment and ensure patients are informed about potential risks, effects, and complications. When cancer therapy is not urgent, patients should visit a dentist at least four weeks before treatment begins. It is crucial for patients to inform their dentists about all medications and cancer treatments to avoid undesirable interactions during dental care. The dentist’s involvement in the oncology care team—providing prevention, early diagnosis, and management of oral complications arising from treatment protocols—significantly enhances cancer treatment success (16).

Teledentistry, defined as the use of health information technologies and telecommunications for oral healthcare delivery, focuses on identifying high-risk populations and has been proposed as an effective tool for remote care, screening, caries detection, diagnosis, consultation, treatment planning, and clinical guidance. It has proven beneficial for remote dental assessment, diagnosis, pain management advice, and provision of interim treatment plans until in-person visits become possible. It also facilitates repetition and reinforcement of patient education between appointments, with notable potential to improve clinical outcomes. The advantage of teledentistry lies in expanding access to dental care; however, further standardized research is needed to fully harness its potential in connecting underserved populations with oral health specialists and promoting optimal oral health (17).

This particular group of oncology patients presents a highly vulnerable profile, as antineoplastic therapies—including chemotherapy, immunotherapy, and targeted therapy—frequently cause numerous, diverse, and sometimes severe oral complications. Under these circumstances, the need for tools enabling systematic, timely, and effective monitoring of oral complications becomes imperative. Teledentistry, with its synchronous and asynchronous communication capabilities, digital applications, and support for personalized instructions, emerges as a promising approach for connecting dentists and oncology patients in real time or through asynchronous remote monitoring. However, despite its theoretical advantages, evidence regarding its clinical effectiveness, acceptance by patients and healthcare professionals, and practical integration into oncology care remains limited (18).

In this context, the present study investigates the effectiveness and applicability of teledentistry models in oncology patients undergoing treatment. The findings aspire to fill a significant gap in the literature and contribute to developing evidence-based recommendations for integrating teledentistry into oncology care. In the Greek healthcare context, where oncology patients often do not receive systematic oral assessment before or during anticancer therapy but rather fragmented and sporadic dental care, the present study suggests that a structured tele-dentistry care model may represent a low-cost, high-effectiveness solution. Such a model would enable hospitals to provide specialized dental support without additional infrastructural burden. Furthermore, our findings reinforce the need to integrate oral health into national cancer control policies (Europe’s Beating Cancer Plan, 2022), as they confirm that oral complications have a direct and measurable impact on overall health and on patients’ ability to continue anticancer treatment. The aim of this study was to investigate the effectiveness of an integrated teledentistry-based dental intervention for patients with solid tumors undergoing antineoplastic therapy, compared with usual oral health care provided by the oncology unit. Specifically, the study examined the role of teledentistry in the prevention, early-stage identification, and monitoring of oral complications, as well as its impact on oral health status and patients’ quality of life.

## Materials and methods

### Study design and setting

This study was designed as a prospective, randomized, interventional clinical trial. It was conducted in two oncology clinics and the outpatient departments of the Oncology Hospital of Kifissia, Athens, Greece, where clinical and radiographic dental examinations and follow-up assessments were performed. The study was approved by the Ethics and Bioethics Committees of the hospital, and all relevant clinical departments were informed. Recruitment and follow-up were carried out over a 21-month period, from March 2022 to November 2023. Participants underwent three clinical and radiographic oral health assessments at baseline, mid-treatment, and end of treatment (T0, T1, and T2).

**Figure 1:**
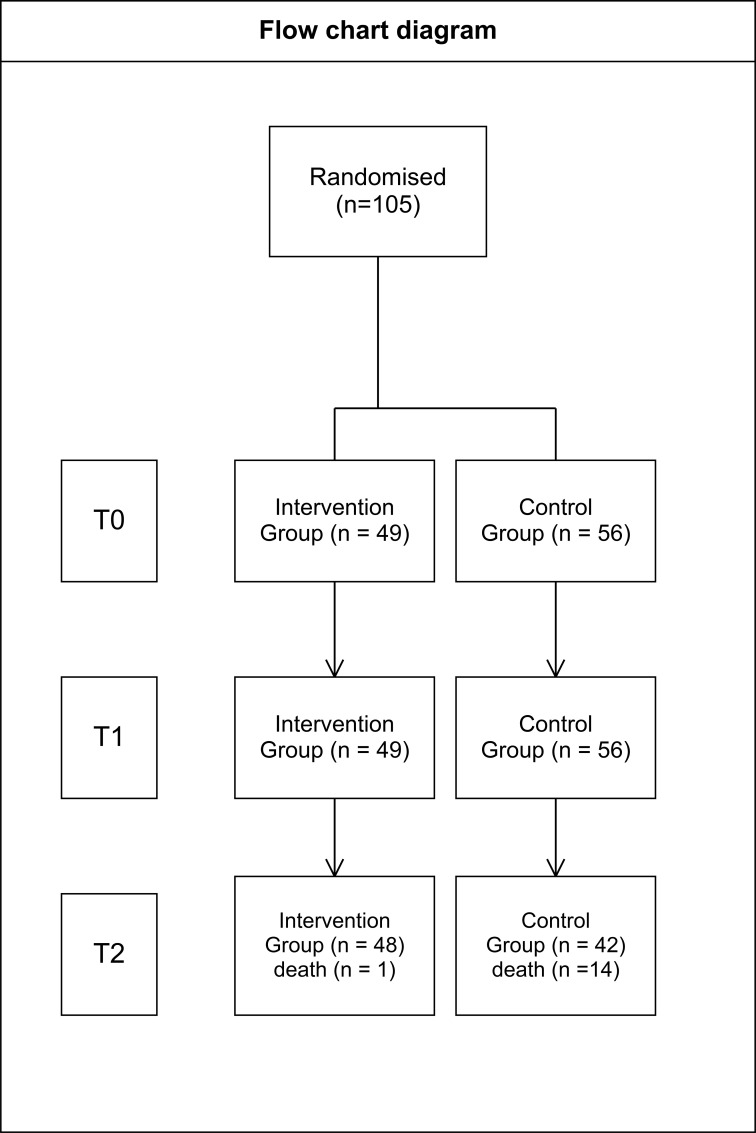
Consort flow diagram.

The study received prior approval from the institutional Ethics and Bioethics Committees of both the participating Oncology Hospital of Kifissia (101/18-02-2022) and the University of West Attica (20256/15-03-2022) before patient recruitment commenced. The trial was not prospectively registered in a public clinical trial registry. Written informed consent was obtained from all participants. Participant flow is presented in a CONSORT flow diagram (**Figure 1**).

### Participants

The study sample consisted of 105 patients with solid tumors of the breast, lung, or colon who presented for the first time for chemotherapy or combination therapy according to a predefined treatment protocol. Eligible patients presenting consecutively to the participating oncology clinics during the recruitment period were invited to participate. Inclusion criteria comprised adult patients with a histologically confirmed diagnosis of a tumor of the breast, lung, or colon who had not previously received chemotherapy. Additional inclusion criteria included an Eastern Cooperative Oncology Group (ECOG) performance status of 0–2, adequate knowledge of the Greek language to ensure informed consent and participation, and basic ability to use a smartphone and/or computer, or cohabitation with a person capable of using such devices.

Exclusion criteria included pre-existing mental or cognitive impairment or other dysfunctional behavioral disorders, poor general health status precluding participation (ECOG 3–4), severe comorbidities associated with intense symptomatology (such as significant physical disability, chronic or life-threatening diseases), age under 18 years, diagnosis of malignancies other than tumors of the breast, lung, or colon, prior initiation of antineoplastic therapy, and inability to understand or communicate in Greek.

### Randomization and allocation

Participants were randomly assigned to intervention or control groups using a computer-generated randomization sequence. Allocation was performed to ensure balanced distribution of key baseline characteristics, including age, gender, and disease stage.

The randomization sequence was generated using a computer-based random number generator by an independent statistician who was not involved in recruitment or clinical assessment. The allocation sequence was stored in a password-protected file accessible only to the independent statistician. Group assignment was communicated to the clinical investigator only after confirmation of baseline data collection (T0), ensuring allocation concealment.

### Outcome measures

Primary outcome measures included the occurrence and severity of oral complications, such as mucosal lesions, gingival bleeding, gingivitis, periodontitis, xerostomia, dental caries, and dysgeusia or dysphagia. Secondary outcomes included oral hygiene behaviors (e.g., brushing frequency), temporomandibular joint dysfunction, and the presence and type of dental prosthetic restorations (fixed or removable).

Baseline demographic and clinical characteristics recorded for group homogeneity included age, gender, educational level, employment status, cancer type and treatment, presence of metastases, surgical history, and functional status according to ECOG (**Table 1**).

**Table 1:** Homogeneity between compared groups.

	Control Group (Instructions)	Intervention Group (Telemedicine)	P-value
Sex; Male/Female	29(51.8)/27(48.2)	22(44.9)/27(55.1)	0.559
**Age**	68.29 ± 10.51	64.86 ± 9.68	0.187
**Educational level;** Elementary/High School/University	24(42.9)/17(30.4)/15(26.8)	12(24.5)/24(49.0)/13(26.5)	0.100
**Marital Status;** Single/Married/Divorced/Widowed	7(12.5)/33(58.9)/11(19.6)/5(8.9)	6(12.2)/30(61.2)/8(16.3)/5(10.2)	0.973
**Family Responsibilities** No/Yes	50(89.3)/6(10.7)	42(85.7)/7(14.3)	0.768
**Employment after Diagnosis;** No/Yes	41(73.2)/15(26.8)	35(71.4)/14(28.6)	1.000
Tumor Site; Breast/Lung/Gastrointestinal	11(19.6)/21(37.5)/24(42.9)	14(28, 6)/10(20.4)/25(51, 0)	0.147
**Metastatic disease;** No/Yes	41(73.2)/15(26.8)	34(69.4)/15(30.6)	0.672
**Surgery performed;** No/Yes	21(37.5)/35(62.5)	15(30.6)/34(69.4)	0.538
**Current Treatment;** Chemotherapy/Combined	30(53.6)/26(46.4)	21(42.9)/28(57.1)	0.271
**ECOG;** 0/1/2/3	3(5.4)/30(53.6)/20(35.7)/3(5.4)	7(14.3)/30(61.2)/12(24.5)/0(0)	0.104
**Toothbrushing;** other/2–3 times per day	46(82, 1)/10(17, 9)	32(65, 4)/17(34, 7)	0.058

Bold values indicate statistically significant differences (p < 0.05).

### Intervention and control procedures

Patients in the control group received standard oral health care according to the usual practice of the oncology unit. They were advised to visit a dentist prior to the initiation of antineoplastic therapy and subsequently managed their oral health according to routine clinical care. Three clinical and radiographic assessments of oral health were performed in both groups during the study, at baseline (before treatment initiation), during treatment, and at the completion of anticancer therapy (T0, T1, and T2).

Patients in the intervention group received a structured, individualized oral health management program delivered by the research dentist. At baseline, patients were educated on oral hygiene practices and instructed on the use of an electronic health platform for reporting oral symptoms. Throughout the study period, participants in the intervention group underwent scheduled teleconsultations via the platform, enabling both synchronous and asynchronous monitoring of oral health between face-to-face clinical evaluations at baseline, mid-treatment, and end of treatment (T0, T1, T2) with assessment timing adapted to the individual oncological treatment protocol.

Oral complications reported through the platform were assessed and managed according to established guidelines from the Multinational Association of Supportive Care in Cancer (MASCC) and the International Society of Oral Oncology (ISOO). In cases requiring in-person evaluation, patients were referred either to the Dental Department of the hospital or to their private dentist, depending on clinical severity. Access to the platform was restricted to the patient and the attending physician to ensure confidentiality.

The intervention followed a predefined standardized protocol applied uniformly to all participants.

### Follow-up and outcome measures

Participants in both study groups underwent three standardized clinical and radiographic oral health assessments at predefined stages of their antineoplastic treatment: prior to treatment initiation (T0), at the midpoint of treatment (T1), and at treatment completion (T2). The timing of these assessments was adapted to each patient’s individual oncological treatment protocol, reflecting differences in cancer type and therapeutic regimen, while maintaining consistent assessment stages across participants.

The primary outcome was the occurrence and severity of oral complications during antineoplastic therapy, as evaluated through clinical and radiographic examination, including mucosal lesions, gingival bleeding, gingivitis, periodontitis, xerostomia, dental caries, and dysgeusia or dysphagia. Secondary outcomes included additional clinical indicators of oral health status, such as oral hygiene–related behaviors (e.g., toothbrushing frequency and technique) and other clinically relevant oral health findings.

Prevention was defined as reduction in oral complication occurrence and severity over time. Early identification was operationally defined as detection of oral mucosal alterations at CTCAE Grade 1, prior to progression to Grade ≥2, during scheduled clinical assessments and monitoring was defined as repeated evaluation across predefined time points (T0–T1–T2).The sample at the end of the treatment decreased by 15 patients due to mortality during antineoplastic treatment (n=15).

### Assessment instruments

Data collection included three structured forms developed by the researcher to record demographic characteristics, general clinical and disease-related information, and findings from the clinical and radiographic oral examination. These data were used to describe baseline characteristics of the study population and to assess comparability between the intervention and control groups at baseline.

In addition, patients completed validated questionnaires assessing quality of life, including the European Organization for Research and Treatment of Cancer Quality of Life Questionnaire Core 30 (EORTC QLQ-C30), the European Organization for Research and Treatment of Cancer Oral Health 15 questionnaire (EORTC QLQ-OH15) and the Hospital Anxiety and Depression Scale (HADS). The EORTC QLQ-C30 and EORTC QLQ-OH15 were used to capture patient-reported outcomes related to general and oral health status, respectively, using standard scoring procedures as defined by the EORTC scoring manuals. The EORTC QLQ-C30 and EORTC QLQ-OH15 were scored according to the official EORTC scoring manuals, with raw scores linearly transformed to a 0–100 scale. For functional scales and global health status, higher scores indicate better functioning, whereas for symptom scales, higher scores indicate greater symptom burden. Psychological distress was assessed using the Hospital Anxiety and Depression Scale (HADS), a14 item instrument measuring symptoms of anxiety and depression in medically ill populations. Permission to use the EORTC questionnaires (EORTC QLQ-C30 and EORTC QLQ-OH15) was obtained from the EORTC Quality of Life Group. Authorization for the use of the Hospital Anxiety and Depression Scale (HADS) was also obtained from the respective copyright holders. The HADS and EORTC questionnaires were administered as part of the broader doctoral research protocol; their analysis will be presented separately.

Periodontal assessment included probing pocket depth (PPD), clinical attachment level (CAL), bleeding on probing (BOP), Gingival Index (GI), and Plaque Index (PI). Measurements were recorded at six sites per tooth using a standardized periodontal probe. Radiographic examination was performed to assess alveolar bone levels and support periodontal diagnosis. All clinical and radiographic examinations were conducted by the same experienced periodontist (study investigator) using standardized diagnostic criteria, the same periodontal probe, and the same dental unit at all assessment time points (T0, T1, T2) to ensure measurement consistency and minimize variability.

Oral mucosal alterations were clinically evaluated using the Oral Mucosa Rating Scale (OMRS), while their severity and functional impact were graded according to the Common Terminology Criteria for Adverse Events (CTCAE v5.0). Xerostomia was assessed based on patient-reported symptoms using predefined clinical criteria. Toothbrushing frequency was recorded as a categorical variable (occasional brushing or 2–3 times daily). Dental caries were assessed through clinical and radiographic examination and classified according to severity as initial, moderate, or advanced lesions based on standardized diagnostic criteria.

### Blinding and bias control

Due to the nature of the intervention, blinding of participants and the research dentist was not feasible. However, several measures were implemented to minimize potential sources of bias. All participants in both study groups underwent standardized clinical and radiographic oral health assessments at predefined stages of treatment (T0, T1, and T2), following the same evaluation protocol. Data collection procedures were standardized, and outcome assessments were based on predefined clinical criteria. Potential sources of bias related to differential intensity of follow-up between groups are acknowledged and addressed in the limitations of the study. All clinical examinations were performed by the same single examiner.

### Statistical analysis

The quantitative and qualitative variables were presented using mean values ± standard deviation and frequencies (percentage) respectively. The Kolmogorov–Smirnov test was used to assess the normality of data distribution. Comparison of homogeneity between the intervention groups in relation to demographic and clinical indicators was performed using the independent samples t-test and the Chi-square test. A Generalized Estimating Equations (GEE) model was used to analyze longitudinal changes in categorical variables between two groups. Generalized Estimating Equations (GEE) models were used to analyze repeated measures of oral health outcomes between compared groups. The model included group (intervention vs. control) as the between-subject factor, time (T0, T1, T2) as the within-subject factor, and the group × time as interaction factor. Results are presented as odds ratios (OR) with 95% confidence intervals (CI) and corresponding p-values. All statistical analyses were performed using the statistical package SPSS version 21.00 (IBM Corporation, Somers, NY, USA). All tests were two-sided. A p-value <0.05 was considered the level of statistical significance.

## Results

A total of 105 eligible patients were enrolled and randomized. Fifteen participants were lost to follow-up between T1 and T2 due to mortality during antineoplastic treatment (14 control, 1 intervention), resulting in 90 participants included in the final analysis. (**Figure 1**).

The effectiveness of the intervention was evaluated through longitudinal changes in oral health outcomes across predefined time points (T0, T1, and T2). Outcomes included mucosal lesions, gingival bleeding, gingivitis, periodontitis, dental caries, temporomandibular (TMJ) disorders and oral hygiene behaviors.

Demographics and clinical characteristics of the study population are summarized in **Table 1**. The two groups were homogeneous at baseline for all variables included gender, age, educational level, marital status and family obligations, employment after diagnosis, type of cancer and type of treatment, presence of metastases, surgical history, and physical activity according to ECOG., with a slight difference in toothbrushing frequency (**Table 1**).

Τhe Instructions group demonstrated a higher probability of mucosal lesions compared with the Telemedicine group at mid-therapy [OR = 2.20 (95% CI: 1.00–4.89), p = 0.053] and a statistically significantly higher probability at the end of therapy [OR = 7.59 (95% CI: 2.98–19.37), p<0.001], while no significant difference was observed at baseline [OR = 1.01 (95% CI: 0.44–2.33), p = 0.977].

The Instructions group demonstrated a statistically significantly higher probability of gingival bleeding compared with the Telemedicine group at mid-therapy [OR = 19.20 (95% CI: 2.44–151.16), p<0.001] and at the end of therapy [OR = 35.25 (95% CI: 4.44–280.15), p<0.001], while no significant difference was observed at baseline [OR = 0.93 (95% CI: 0.43–2.02), p = 0.853].

The Instructions group demonstrated a statistically significantly higher probability of gingivitis compared with the Telemedicine group at mid-therapy [OR = 20.92 (2.67-164.25), p<0.001] and at the end of therapy [OR = 38.83 (4.89-308.25), p<0.001], while no significant difference was observed at baseline [OR = 0.93 (0.43-2.02), p=0.853].

The Instructions group demonstrated a statistically significantly higher probability of dental caries compared with the Telemedicine group at mid-therapy [OR = 3.12 (1.12-8.72), p=0.030] and at the end of therapy [OR = 26.11 (3.27-208.76, p<0.001], while no significant difference was observed at baseline [OR = 0.93 (0.43-2.02), p=0.853].

No statistically significant difference between the groups was found at baseline [OR = 1.21 (0.55-2.70), p=0.627] mid-treatment [OR = 1.58 (0.70-3.54) p=0.267] or at the end of treatment [OR = 2.04 (0.83-5.00), p=0.120] in the evaluation of temporomandibular joint disorders (TMJDs) (**Table 2**).

**Table 2:** Comparison of dental problems between groups.

	Groups	Baseline (T0)	Mid-therapy (T1)	End of therapy (T2)
		(No/Yes)	(No/Yes),	(No/Yes)
Mucosal Lesions	Telemedicine Intervention group	15(30.6)/34(**69.4**)	24(49.0)/25(**51.0**)	35(72.9)/13(**27.1**)
	Instructions Control group	16(30.4)/40(**71, 4**)	17(30.4)/39(**69.6**)	11(26.2)/31(**73.8**)
	OR(95% CI) p-value	1.01(0.44-2.33) p=0.977	2.20 (1.00-4.89) **p=0.053**	7.59 (2.98-19.37) **p<0.001**
Gingival Bleeding	Telemedicine Intervention group	28(57.1)/21(**42.9**)	49(100.0)/0(**0.0**)	48(100.0)/0(**0.0**)
	Instructions Control group	33(58.9)/23(**41.1**)	40(71.4)/16(**28.6**)	24(57.1)/18(**42.9**)
	OR(95% CI) p-value	0.93 (0.43-2.02) p=0.853	19.20 (2.44-151.16) **p<0.001**	35.25 (4.44-280.15) **p<0.001**
Gingivitis	Telemedicine Intervention group	28(57.1)/21(**42.9**)	48(98.0)/1(**2.0**)	48(100.0)/0(**0.0**)
	Instructions Control group	33(58, 9)/23(**41.1**)	39(69.6)/17(**30.4**)	23(54, 8)/19(**45.2**)
	OR(95% CI) p-value	0.93 (0.43-2.02) p=0.853	20.92 (2.67-164.25) **p<0.001**	38.83 (4.89-308.25) **p<0.001**
Dental Caries	Telemedicine Intervention group	33(68.8)/16(**32.7**)	43(87.8)/6(**12.2**)	47(97.9)/1(**2.1**)
	Instructions Control group	38(67.9)/18(**32.1**)	39(69.6)/17(**30.4**)	27(64.3)/15(**35.7**)
	OR(95% CI) p-value	0.98 (0.43-2.22) p=0.956	3.12 (1.12-8.72) **p=0.030**	26.11 (3.27-208.76) **p<0.001**
Temporomandibular (TMJ) Disorders	Telemedicine Intervention group	32(65.3)/17(**34.7**)	34(69.4)/15(**30.6**)	36(75)/12(**25**)
	Instructions Control group	34(60.7)/22(**39.3**)	33(58.9)/23(**41.1**)	25(59.5)/17(**40.5**)
	OR(95% CI) p-value	1.21 (0.55-2.70) p=0.627	1.58 (0.70-3.54) p=0.267	2.04 (0.83-5.00) p=0.120

Bold values indicate statistically significant differences (p < 0.05).

The Instructions group demonstrated a statistically significantly higher probability of moderate or advanced periodontitis compared with the Telemedicine group at mid-therapy [OR = 3.63 (1.52-8.67), p<0.001] and at the end of therapy [OR = 8.25 (2.50-27.18), p<0.001], while no significant difference was observed at baseline [OR = 1.63 (0.74-3.59) p=0.224].

The Telemedicine group demonstrated a statistically significantly higher probability of brushing 2–3 times per day compared with the Instructions group at mid-therapy [OR = 3.60 (1.45-8.96), p=0.006] and at the end of therapy [OR = 5.52 (1.96-15.51), p<0.001], while no significant difference was observed at baseline [OR = 2.44 (1.00-6.03), p=0.058] (**Table 3**).

**Table 3:** Comparison of dental problems between groups (continued).

	Groups	Baseline (T0)	Mid-therapy (T1)	End of therapy (T2)
		(No/Early/Moderate/Advanced)	(No/Early/Moderate/Advanced)	(No/Early/Moderate/Advanced)
Periodontitis	Telemedicine Intervention group	32(65.3)/17(**34.7**)	39(79.6)/10(**20.4**)	44(91.7)/4(**8.3**)
	Instructions Control group	30(53.6)/26(**46.4**)	29(51.8)/27(**48.2**)	24(57.1)/18(**42.9**)
	p-value	1.63 (0.74-3.59) p=0.224	3.63 (1.52-8.67) **p<0.001**	8.25 (2.50-27.18) **p<0.001**
		Rarely or once per day /2–3 times per day	Rarely or once per day /2–3 times per day	Rarely or once per day /2–3 times per day
Toothbrushing Frequency	Telemedicine Intervention group	32(65.4)/17(**34.7**)	29(59.2)/20(**40**.8)	25(52.1)/23(**47.9**)
	Instructions Control group	46(82.1)/10(**17.9**)	47(83.9)/9(**16.1**)	36(85.7)/6(**14.3**)
	p-value	2.44 (1.00-6.03) p=0.058	3.60 (1.45-8.96) **p=0.006**	5.52 (1.96-15.51) **p<0.001**

Bold values indicate statistically significant differences (p < 0.05).

A complete-case sensitivity analysis was performed including only participants with complete follow-up data. The results were consistent with the primary GEE analysis and did not materially alter the study conclusions (**Table 4**).

**Table 4:** Comparison of dental problems between groups (only participants with complete data were included).

	Groups	Baseline (T0)	Mid-therapy (T1)	End of therapy (T2)
		(No/Yes)	(No/Yes)	(No/Yes)
Mucosal Lesions	Telemedicine (reference) ν=48	1.28(0.51-3.21) p=0.600	2.32 (1.22-3.95) **p=0.008**	5.92 (2.77-12.64) **p<0.001**
	Control ν=42			
Gingival Bleeding	Telemedicine (reference) ν=48	1.05(0.45-2.43) p=0.909	17.91(1.91-168.21) **p=0.012**	33.57(3.61-311.90) **p=0.002**
	Control ν=42			
Gingivitis	Telemedicine (reference) ν=48	1.05(0.45-2.43) p=0.909	20.10(2.53-159.33) **p=0.005**	37.00(4.02-340.33) **p=0.001**
	Control ν=42			
Dental Caries	Telemedicine (reference) ν=48	1.23(0.52-2.92) p=0.638	3.16(1.33-7.48) **p=0.009**	21.22 (2.88-156.49) **p=0.003**
	Control ν=42			
Temporomandibular (TMJ) Disorders	Telemedicine (reference) ν=48	1.12(0.48-2.65) p=0.793	1.33(0.88-2.01) p=0.172	1.82 (0.93-2.54) p=0.079
	Control ν=42			
		No-Early/Moderate-Advanced	No-Early/Moderate-Advanced	No-Early/Moderate-Advanced
Periodontitis	Telemedicine (reference) ν=48	1.65 (0.70-3.88) p=0.249	2.17 (1.26-3.73) **p=0.005**	5.02 (1.92-13.02) **p=0.001**
	Control ν=42			
		Rarely or once per day /2–3 times per day	Rarely or once per day /2–3 times per day	Rarely or once per day /2–3 times per day
Toothbrushing Frequency	Control (reference) ν=42	2.55 (0.99-6.49) p=0.055	3.57(1.32-9.65) **p=0.012**	5.35(1.88-14.88) **p=0.001**
	Telemedicine ν=48			

All analyses were presented as OR(95%CI), OR, Odds Ratio; CI, Confidence Interval.

Bold values indicate statistically significant differences (p < 0.05).

**Figure 2:**
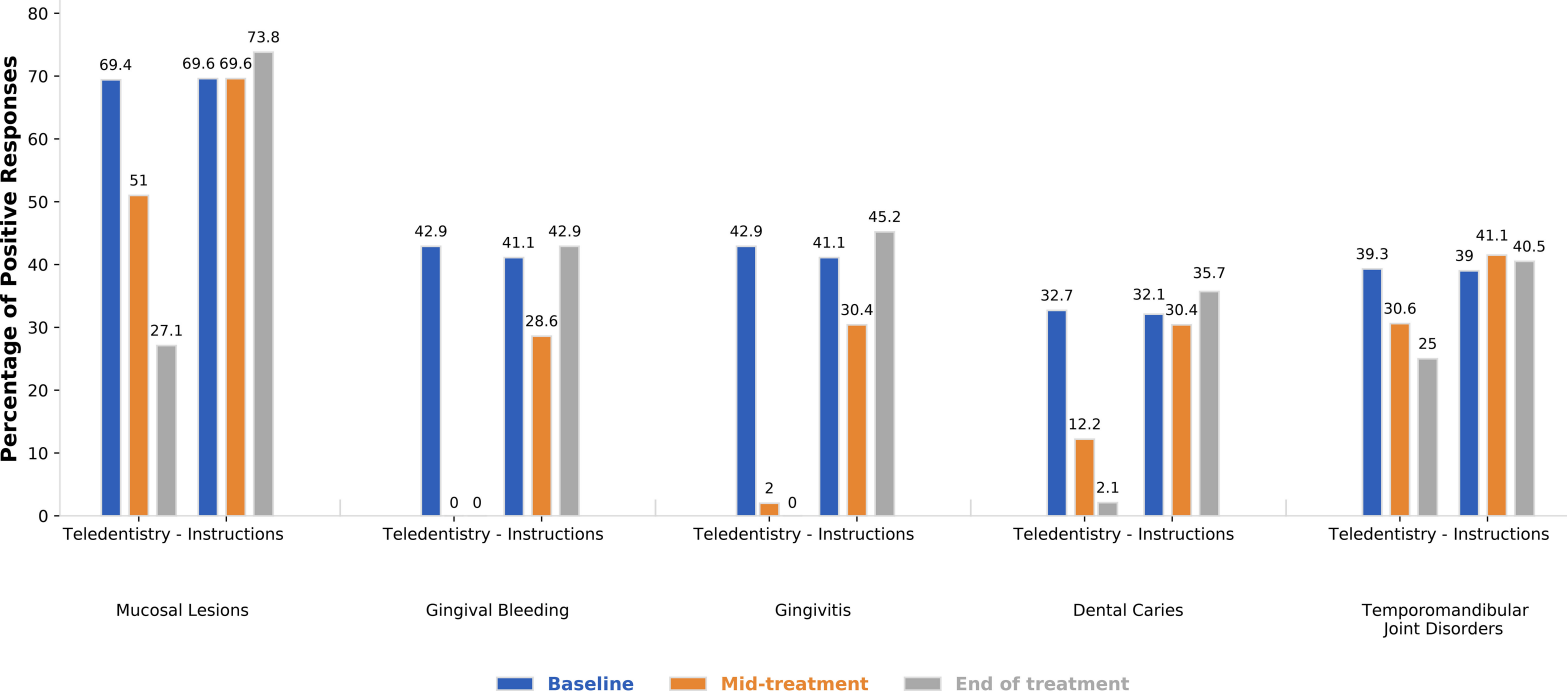
Longitudinal comparison of dental problems between groups.

Adherence to study procedures was assessed in both groups. In the control group (n = 56), participants received standard advice to visit a dentist at baseline and were reassessed during treatment and at its completion. Twelve participants (21.4%) reported having visited a dentist during the study period. In the intervention group (n = 49), a total of 147 remote communications were scheduled during the follow-up period and 135 were of these were completed, corresponding to an adherence rate of 91.8%. Fourteen participants (28.6%) reported oral symptoms through the remote monitoring process, six participants (12.2%) were referred for dental evaluation, and all referred patients subsequently attended a dental visit (6/6, 100%). **Figure 2** presents the longitudinal evolution of oral health outcomes across the three assessment time points (T0, T1, and T2) in the intervention and control groups. During cancer therapy, the intervention group consistently exhibited statistically significant lower rates of mucosal lesions, gingival bleeding, gingivitis, dental caries, and periodontitis, as well as higher toothbrushing frequency compared with the control group.

These findings demonstrate longitudinal changes in oral health outcomes across treatment stages (**Figure 2**).

## Discussion

The management of oral complications in patients undergoing chemotherapy or combined anticancer treatments is the subject of extensive international guidelines and protocols. Despite some differences among countries and organizations, most protocols converge on several fundamental principles of prevention, monitoring, and management of these complications.

Within the context of investigating the impact of oral complications on oncology patients, the findings of the present study were compared with recent international primary studies. Our results demonstrated that the intervention group of structured oral monitoring exhibited a lower percentage of mucosal lesions compared with the control group at mid-treatment (51% vs 69.6%) and at the end of antineoplastic therapy (27.1% vs 73.8%).

In a 6-month prospective study in adult patients with solid tumors, Padure et al. (2024), reported the frequency and severity of oral mucositis, and identified specific preventive factors associated with reduced complication severity. These findings are consistent with our results, suggesting that structured and systematic oral-health monitoring during cancer therapy may contribute to improved clinical outcomes. In our study, the telemedicine-supported follow-up facilitated continuous supervision and structured clinical management of reported oral complications (19).

The incidence of oral mucositis observed in our cohort is consistent with previous reports indicating that 30–40% of patients receiving chemotherapy without radiotherapy develop oral mucositis, with substantially higher rates reported in head and neck cancer patients undergoing combined chemoradiotherapy (20).

Emerging evidence suggests that chemotherapy-induced alterations of the oral microbiome may contribute to mucosal inflammation and lesion severity. Mucosal lesions in the present cohort frequently co-occurred with other oral alterations, highlighting the dynamic interaction between systemic treatment and oral health status. These findings reinforce the clinical importance of proactive oral assessment before treatment initiation and continuous monitoring throughout the course of antineoplastic therapy (21–23).

In the present study, statistically significant differences between groups were observed at mid-treatment and treatment completion for gingival bleeding, gingivitis, periodontitis, and dental caries, with the intervention group consistently demonstrating lower rates of deterioration compared with the control group. In contrast, no significant differences were found at baseline, confirming initial comparability between groups. Notably, toothbrushing frequency increased significantly in the intervention group over time, whereas the control group showed limited behavioral change.

The progressive worsening of gingival bleeding and periodontitis observed in the control group during antineoplastic therapy is consistent with previous evidence indicating that chemotherapy is associated with microbial imbalance and increased periodontal vulnerability. Soutome et al. (2021) described a higher pathogenic bacterial load in the oral microbiota during treatment. Vozza et al. (2015) also reported early gingival inflammation and periodontal sensitivity from the first treatment cycles. Sharma and Singh (2020) further highlighted the association between chemotherapy, periodontal inflammation, and elevated inflammatory markers (24–26).

Importantly, in our study, patients receiving structured telemonitoring demonstrated stabilization or improvement of periodontal parameters across treatment stages. Collectively, these findings are consistent with the view that structured monitoring and reinforced oral hygiene may help limit periodontal deterioration during chemotherapy.

Furthermore, the significant increase in toothbrushing frequency observed in the intervention group and its association with improved gingival outcomes align with the findings of Parreiras et al. (2024), who reported that reduced oral hygiene practices were associated with increased gingival bleeding and poorer oral-health-related quality of life. Additional evidence from Flores et al. (2025) and Poulopoulos et al. (2017) underscores the clinical importance of maintaining oral health during oncologic therapy, as caries and oral discomfort directly affect patients’ daily functioning and overall well-being (27–29).

The systematic review by Hong et al. (2019) and the meta-analysis by Moussa et al. (2025) document that the implementation of structured preventive oral care protocols significantly reduces inflammatory lesions, gingival bleeding, and other oral side effects during cancer therapy, contributing to improved clinical outcomes (30, 31). In agreement with these reports, our randomized intervention, which incorporated structured telemonitoring and oral-hygiene guidance, was associated with significantly lower rates of mucosal lesions, gingival bleeding, gingivitis, periodontitis, and caries at mid-treatment and treatment completion compared with usual care. These results further support the clinical value of implementing systematic and protocol-driven oral-health management in oncology settings.

Another important element supporting our observations is the relationship between mucositis and alterations in the oral microbiome. Studies have shown that chemotherapy induces bacterial dysbiosis, with a decrease in healthy communities such as Streptococcus, Actinomyces, and an increase in pathogenic Gram-negative bacteria, which correlates with the severity of mucositis. Our finding that many of our patients developed mucositis alongside other lesions underlines the importance of strengthened preventive and daily oral-care approaches — a conclusion strongly supported by the international literature (24, 26).

Clinically, our results underscore the importance of preventive dental assessment before the initiation of chemotherapy, as well as continuous oral monitoring throughout all treatment cycles.

In our cohort, most prosthetic rehabilitations were fixed restorations, while nineteen patients wore complete removable dentures. Although prosthesis type was not analyzed as an independent variable, oral candidiasis was descriptively observed among denture wearers. Given the known predisposition to Candida colonization in complete denture users, particularly under immunosuppressive conditions, this observation underscores the need for individualized preventive monitoring (32).

In our study, no significant effects were observed on the temporomandibular joint (TMJ) of patients undergoing anti-neoplastic treatments. This finding seems to differ from some reports in the international literature, which point out that both chemotherapy and radiotherapy can affect the musculoskeletal system and joints, including the temporomandibular joint. However, according to the UK Medicines Information (2019), these effects are not consistently documented. In practice, joint involvement seems to vary depending on treatment type, dose, duration, and individual patient factors. This variability may partly explain why we did not observe significant TMJ changes in our sample (29). Furthermore, the study by Calik et al. (2024) highlights that the use of bisphosphonates and the presence of bone metastases have a greater impact on oral health and possibly on jaw stability, than on direct changes in the TMJ from the antineoplastic therapy (33).

The present study highlights the importance of organized, systematic monitoring of the oral health of oncology patients. In our intervention, telemonitoring was implemented through scheduled remote consultations, structured symptom reporting via a digital platform, and direct communication with a dental specialist. This structured follow-up enabled structured clinical evaluation of reported symptoms and facilitated management according to established guidelines. This is particularly crucial for conditions such as mucositis, where intervention at low grade severity reduces pain and the need for hospitalization, or candidiasis, which can easily be overlooked without regular assessment (34–36).

Telemonitoring in our study rather than replacing conventional clinical evaluation, functioned as an additional layer of supervision, helping to sustain preventive behaviors and allowing guideline based clinical response when complications emerged during antineoplastic therapy.

### Limitations

As with most clinical research in oncology, some study-related factors should be taken into account. Attrition occurred between T1 and T2 due to mortality during antineoplastic treatment (n = 15), which is expected in an oncology population undergoing systemic therapy. Although this reduced the final sample size, the overall longitudinal trends remained consistent between assessment points. During follow-up, more deaths occurred in the control group (14/56) than in the intervention group (1/49), likely reflecting the progression of the underlying malignancy during antineoplastic therapy. Mortality was not a predefined study outcome. A sensitivity analysis excluding deceased participants yielded results consistent with the primary analysis, suggesting that this imbalance did not materially affect the study findings.

Also, due to the nature of the intervention, blinding of participants and the clinical examiner was not feasible. However, standardized clinical and radiographic protocols were applied at all time points to ensure consistency of measurements.

In addition, the intervention group received more structured follow-up compared with usual care. Therefore, the observed benefit reflects the effect of a comprehensive telehealth-supported oral care model rather than digital monitoring alone.

Finally, initial difficulty in the use of the telemedicine platform was observed in some participants; however, this was mitigated through structured guidance and repeated instruction.

### Strengths

The present study has several methodological and clinical strengths. It was designed as a prospective randomized controlled trial with longitudinal assessment at three predefined time points (T0–T1–T2), enabling evaluation of temporal changes in oral-health outcomes during active antineoplastic therapy. To our knowledge, this is the first prospective randomized study conducted in Greece to comprehensively investigate oral health in oncology patients with solid tumors (breast, colorectal, and lung cancer) during active antineoplastic treatment, and among the limited studies internationally employing systematic clinical and radiographic follow-up in this population.

Combined with its innovative design, the present study provides novel and robust evidence supporting the implementation of a structured and multidisciplinary oral-health monitoring model for oncology patients. A central methodological strength lies in the integration of structured telemedicine-based monitoring within routine oncology care, enabling continuous, protocol-driven surveillance of oral complications throughout treatment rather than isolated or fragmented clinical assessments.

Also, oral-health status was evaluated through systematic clinical and radiographic examinations performed by a single calibrated examiner using the same dental unit, enhancing diagnostic consistency and minimizing measurement variability. Moreover, the study further combined objective clinical endpoints with behavioral indicators, such as toothbrushing frequency, allowing a comprehensive assessment of both clinical effectiveness and patient adherence. Another important strength of the study lies in its implementation within the demanding workflow of a tertiary oncology hospital. All clinical and radiographic examinations were conducted during routine outpatient clinic hours, requiring careful coordination within existing infrastructure and without dedicated dental facilities. This real-world integration enhances the external validity and feasibility of the proposed monitoring model.

The digital monitoring framework facilitated real-time follow-up during active oncological treatment and supported identification and management of oral complications, thereby strengthening continuity of care in a population in which systematic dental surveillance is not implemented. The findings may therefore have broader implications in public health for the integration of telemedicine-based oral-health monitoring into supportive oncology care.

### Suggestions for clinical practice and health policy

In Greece, although clinical guidelines have been issued by scientific societies such as the Hellenic Society of Head and Neck Oncology (HeSHNO), the implementation of systematic oral-care protocols in hospital settings remains limited. The integration of a dedicated dental specialist into the oncology care team could address this gap by providing individualized and evidence-based oral-health care. Continuous oral-health monitoring may reduce the risk of severe complications and may help ensure uninterrupted cancer therapy.

Our study demonstrated that the combined use of in-person and remote monitoring offers substantial benefits in both the prevention and management of oral complications, while also enhancing patient adherence. Given the demonstrated benefits of teledentistry, its integration should be promoted across multiple levels of oral-health services, including interpersonal, organizational, and national levels. Teledentistry offers important social benefits for underserved populations, as well as advantages in time efficiency and cost reduction.

Although the use of digital health technologies may initially appear challenging for some patients, these barriers can be effectively addressed through brief training, leading to high levels of acceptance and satisfaction. Data transmission methods, connection speed, and data management must be handled carefully and responsibly to ensure effectiveness and maintain trust in the use of available technologies. With multiple benefits for both dental professionals and patients, teledentistry represents a highly promising and increasingly relevant tool in contemporary dental practice.

## Conclusions and future directions

In conclusion, the observed oral complications in oncology patients highlight the significant role of oral health in the overall course of anticancer therapy in oncology patients and the development of local infections indicate the significant role of the oral cavity in the emergence of systemic complications during anticancer therapy, as well as the need for vigilance and continuous monitoring of the mouth. The timely recognition, diagnosis, and management of oral mucosal complications throughout cancer treatment are critical for administering and completing therapy within the scheduled timeframe, thereby achieving the best possible therapeutic outcome, reducing morbidity, and preserving the quality of life of the already burdened cancer patient.

This clinical study supports the integration of systematic and comprehensive dental care as an important component of oncological management. Overall, international standards highlight the importance of interdisciplinary collaboration and the integration of oral care into oncological follow-up, combining prevention, early diagnosis, and individualized management of oral complications. A protocol of this type, which we propose, could include:

1. Pre-treatment dental assessment and management of oral conditions.
2. Daily oral care instructions for patients and caregivers.
3. Regular evaluation using standardized tools, with monitoring frequency adapted to the treatment regimen.
4. Intervention guidelines in cases of mucositis or other complications.
5. Integration of telemedicine/e-Health applications for continuous monitoring and patient education.

The combined use of both in-person and remote monitoring via e-health applications offers substantial benefits for both the prevention and management of oral complications, enhancing patient compliance and supporting overall oral health during antineoplastic therapy.

## Data Availability

The original contributions presented in the study are included in the article/supplementary material. Further inquiries can be directed to the corresponding author.
